# Efficacy and safety of ridinilazole compared with vancomycin for the treatment of *Clostridium difficile* infection: a phase 2, randomised, double-blind, active-controlled, non-inferiority study

**DOI:** 10.1016/S1473-3099(17)30235-9

**Published:** 2017-07

**Authors:** Richard J Vickers, Glenn S Tillotson, Richard Nathan, Sabine Hazan, John Pullman, Christopher Lucasti, Kenneth Deck, Bruce Yacyshyn, Benedict Maliakkal, Yves Pesant, Bina Tejura, David Roblin, Dale N Gerding, Mark H Wilcox, CoDIFy study group, Amit Bhan, Wayne Campbell, Teena Chopra, Kenneth Deck, Yoav Golan, Ian Gordon, Ravi Kamepalli, Sahil Khanna, Christine Lee, Christopher Lucasti, Benedict Maliakkal, Irene Minang, Kathleen Mullane, Richard Nathan, Matthew Oughton, Yves Pesant, John Phillips, John Pullman, Paul Riska, Christian Schrock, Jonathan Siegel, Alon Steinberg, David Talan, Stephen Tamang, Michael Tan, Karl Weiss, Chia Wang, Bruce Yacyshyn, Jo-Anne Young, Jonathan Zenilman

**Affiliations:** a Summit Therapeutics, Abingdon, UK; b GST Micro, Durham, NC, USA; c Idaho Falls Infectious Diseases, Idaho Falls, ID, USA; d Ventura Clinical Trials, Ventura, CA, USA; e Mercury Street Medical, Butte, MT, USA; f South Jersey Infectious Disease, Somers Point, NJ, USA; g Alliance Research Centers, Laguna Hills, CA, USA; h University of Cincinnati, Cincinnati, OH, USA; i University of Rochester Medical Center, Rochester, NY, USA; j St-Jerome Medical Research, St-Jérôme, QC, Canada; k Edward Hines Jr Veterans Administration Hospital and Loyola University Chicago, Stritch School of Medicine, Maywood, IL, USA; l Microbiology, Leeds Teaching Hospitals and University of Leeds, Leeds, UK

## Abstract

**Background:**

*Clostridium difficile* infection is the most common health-care-associated infection in the USA. We assessed the safety and efficacy of ridinilazole versus vancomycin for treatment of *C difficile* infection.

**Methods:**

We did a phase 2, randomised, double-blind, active-controlled, non-inferiority study. Participants with signs and symptoms of *C difficile* infection and a positive diagnostic test result were recruited from 33 centres in the USA and Canada and randomly assigned (1:1) to receive oral ridinilazole (200 mg every 12 h) or oral vancomycin (125 mg every 6 h) for 10 days. The primary endpoint was achievement of a sustained clinical response, defined as clinical cure at the end of treatment and no recurrence within 30 days, which was used to establish non-inferiority (15% margin) of ridinilazole versus vancomycin. The primary efficacy analysis was done on a modified intention-to-treat population comprising all individuals with *C difficile* infection confirmed by the presence of free toxin in stool who were randomly assigned to receive one or more doses of the study drug. The study is registered with ClinicalTrials.gov, number NCT02092935.

**Findings:**

Between June 26, 2014, and August 31, 2015, 100 patients were recruited; 50 were randomly assigned to receive ridinilazole and 50 to vancomycin. 16 patients did not complete the study, and 11 discontinued treatment early. The primary efficacy analysis included 69 patients (n=36 in the ridinilazole group; n=33 in the vancomycin group). 24 of 36 (66·7%) patients in the ridinilazole group versus 14 of 33 (42·4%) of those in the vancomycin group had a sustained clinical response (treatment difference 21·1%, 90% CI 3·1–39·1, p=0·0004), establishing the non-inferiority of ridinilazole and also showing statistical superiority at the 10% level. Ridinilazole was well tolerated, with an adverse event profile similar to that of vancomycin: 82% (41 of 50) of participants reported adverse events in the ridinilazole group and 80% (40 of 50) in the vancomycin group. There were no adverse events related to ridinilazole that led to discontinuation.

**Interpretation:**

Ridinilazole is a targeted-spectrum antimicrobial that shows potential in treatment of initial *C difficile* infection and in providing sustained benefit through reduction in disease recurrence. Further clinical development is warranted.

**Funding:**

Wellcome Trust and Summit Therapeutics.

## Introduction

*Clostridium difficile* infection is the most common hospital-acquired infection in the USA,^1^ with an estimated 453 000 incident cases reported in the country in 2011, accounting for 29 300 deaths.^2^ A pan-European study estimated the mean incidence of hospital-acquired *C difficile* infection to have increased by 71% from 4·1 to 7·0 cases per 10 000 patient-days between 2008 and 2012–2013.^3^, ^4^ An estimated 40 000 diagnoses are missed each year in hospitals in Europe because of a lack of clinical suspicion and suboptimal diagnostic methods.^3^

Patients with *C difficile* infection have significantly higher inpatient mortality, spend longer periods in intensive care, and have higher rates of all-cause readmission over 3 months than do matched controls.^5^ In Europe, a systematic review^6^ of data from 14 countries revealed 30-day crude mortality as high as 29·8%, with rates doubling between 1994 and 2004. The economic burden of *C difficile* infection is also substantial, with higher total costs of hospital admission and treatment in patients with *C difficile* infection than in controls.^5^

Recurrence, resulting from relapse of the initial infection or reinfection with a new strain of *C difficile,* occurs in up to 30% of patients following initial therapy.^7^, ^8^ The risk of further recurrence increases appreciably with each episode, increasing to approximately 45% in patients with one previous episode and to approximately 65% following a second recurrence.^9^ Recurrent *C difficile* infection places a substantial burden on patient welfare, is associated with increased morbidity and mortality, and is difficult to treat.^10^

**Research in context**

**Evidence before this study**

A search of PubMed was done with the terms “ridinilazole” or “SMT19969”, with no other restrictions on terms, and limited to a 5-year period before approval of the protocol (Dec 1, 2008, to Dec 1, 2013). The search identified two articles describing the in-vitro activity of ridinilazole showing potent growth inhibition of *Clostridium difficile* and that ridinilazole possessed a targeted spectrum of activity, with little or no growth inhibition of either Gram-positive or Gram-negative members of the intestinal microbiota. Additional evidence was available to the authors at the time, including results from a phase 1 study showing ridinilazole to be safe, well tolerated, localised to the gastrointestinal tract, and to largely preserve the intestinal microbiota of the trial participants. Non-clinical data included results from the hamster model of *C difficile* infection, which showed that ridinilazole protected against both acute infection and disease recurrence. These data have subsequently been published. On the basis of this evidence, it was decided to assess the efficacy and safety of ridinilazole in a phase 2 clinical trial.

**Added value of this study**

This phase 2, randomised, double-blind, active-controlled study shows the non-inferiority of ridinilazole compared with vancomycin in terms of sustained clinical cure of *C difficile* infection (ie, clinical cure at the end of treatment and no recurrence within 30 days), while being safe and well tolerated.

**Implications of all available evidence**

Ridinilazole has potential as a new treatment of *C difficile* infection, owing to its good sustained clinical response rates compared with those of conventional therapy, probably as a result of decreased disturbance of the normally protective intestinal microbiota. The findings of our study support further clinical development of ridinilazole.

Three antibiotics are available for treatment of *C difficile* infection: metronidazole, vancomycin, and fidaxomicin. Metronidazole is only recommended for treatment of mild-to-moderate episodes^11^, ^12^ and has been shown to be inferior to vancomycin.^13^ Both metronidazole and vancomycin are associated with high rates of recurrence due to disruption of the normal microbiota during therapy.^14^ Fidaxomicin is non-inferior to vancomycin with respect to clinical response at the end of treatment but superior with regard to sustained clinical response up to 25 days after cessation of treatment.^7^, ^8^ However, when compared with vancomycin, fidaxomicin does not improve sustained clinical responses against BI/NAP1/027 strains.^7^, ^8^ These observations underscore the need for safe and effective alternatives that do not negatively affect the normal gut microbiota, thereby potentially facilitating prevention of recurrent *C difficile* infection.

Ridinilazole (formerly known as SMT19969) is an antimicrobial restricted to the gastrointestinal tract. In-vitro studies have shown its high inhibitory activity against *C difficile* and minimal activity against both Gram-positive and Gram-negative aerobic and anaerobic intestinal microorganisms,^15^, ^16^, ^17^, ^18^, ^19^ and studies in a hamster model of *C difficile* infection have shown the effectiveness of ridinilazole in prevention of both primary and recurrent episodes of infection.^20^ In a phase 1 study,^21^ ridinilazole was safe and well tolerated in healthy human volunteers, with little systemic absorption and little effect on normal gut microbiota following administration of single or multiple doses.

This phase 2 trial was designed to assess the efficacy and safety of ridinilazole compared with vancomycin for the treatment of *C difficile* infection in adults.

## Methods

### Study design

This randomised, double-blind, active-controlled study was done at 33 centres, primarily hospitals and out-patient clinics, in the USA and Canada. Participants were recruited between June 26, 2014, and August 31, 2015. We did a preliminary assessment of all patients with a positive diagnostic test for *C difficile* infection, on the basis of protocol inclusion and exclusion criteria, to determine potential suitability for the study before approaching them for formal screening for enrolment into the study. The appendix shows further details of study design and participants. Ethics approval was obtained from the institutional review board at each centre. The study adhered to ethical principles as set forth in the Declaration of Helsinki and followed all principles of good clinical practice.

### Participants

This study included men and women aged 18–90 years. Eligible participants had *C difficile* infection (defined as more than three unformed bowel movements or more than 200 mL of unformed stool in rectal collection devices 24 h before randomisation) and a positive local diagnostic test for *C difficile* infection (detection of either a toxigenic strain by nucleic acid amplification tests or free toxin in stool by enzyme immunoassay). Participants could have received no more than 24 h of antimicrobial treatment with metronidazole, vancomycin, or fidaxomicin before initiation of the study drug and were either treated as inpatients or as outpatients for part or all of the study. All participants provided written informed consent.

### Randomisation and masking

Participants were randomly assigned in a 1:1 ratio to receive either ridinilazole or vancomycin. Stratification was done on the basis of centre, age (>75 years and ≤75 years), history of recurrent *C difficile* infection over the past 12 months (no occurrences and one to three previous occurrences), and presence of *C difficile* toxin A (TcdA) or toxin B (TcdB) at screening (either free toxin positive or free toxin negative and free toxin not tested at screening). Stratified randomisation was done by use of a dynamic allocation procedure (MedPace, Cincinnati, OH, USA). No participant could begin treatment before receiving a unique randomisation number, which was created by an allocation-concealed, computer-generated random permutation procedure. The investigators enrolled participants into the trial. Randomisation and study group assignment was done by an interactive voice and web response system (IVRS/IWRS) overseen by MedPace. Participants, investigators, and other study personnel remained blinded to the treatment received until the database was locked, except if an unblinding procedure was needed (appendix).

Blinding was achieved by over-encapsulation of both study drugs (ridinilazole and vancomycin) and a placebo within identical size zero, Swedish orange, hard gelatin immediate-release capsules.

### Procedures

Ridinilazole (200 mg) was given orally twice daily for 10 days, with participants also receiving two doses of placebo to preserve blinding through consistency with the dosing schedule in the vancomycin group, while vancomycin (125 mg) was given orally four times daily for 10 days. Use of other antibiotics to treat *C difficile* infection was prohibited, as were probiotics, antidiarrhoeal drugs, antiperistaltic drugs, and other products used to slow bowel movement. The first dose of study medication was administered following completion of screening procedures, baseline assessments, and randomisation (appendix). As part of the baseline assessments, stool samples were assessed for the presence of free *C difficile* toxin with either an enzyme immunoassay test (C. Diff Quik Chek Complete; Alere, Waltham, MA, USA) provided by the sponsor to each site or a cell cytotoxicity neutralisation assay at a central laboratory (South Bend Medical Foundation, South Bend, IN, USA). Participants were assessed for clinical response to treatment on day 4–6, at the end of treatment (day 10–11), at test of cure (day 12–14), at weekly follow-up visits after completion of treatment (day 13–39), at a follow-up site visit (day 22–28), and at an end-of-study visit (day 37–43). In addition, unscheduled visits could be made if recurrence was suspected.

### Outcomes

The primary objective of the study was to assess the efficacy of ridinilazole compared with vancomycin in the treatment of *C difficile* infection. The primary efficacy endpoint was sustained clinical response, defined as clinical cure (less than or equal to three unformed bowel movements in a 24-h period or less than 200 mL unformed stool in rectal collection devices) at test of cure and no recurrence of *C difficile* infection within 30 days after end of treatment. Recurrence was defined as a new episode of diarrhoea between test of cure and end of the study, resulting in the individual receiving antimicrobial treatment for *C difficile* before the end of the study. We followed up patients for recurrence for 30 days after the end of treatment, in keeping with similar phase 2 and phase 3 clinical trials.^7^, ^8^, ^22^ Secondary efficacy endpoints were clinical cure at test of cure (ie, at day 12–14), time to resolution of diarrhoea (defined as the time from starting study drug to the first formed bowel movement not followed by an unformed bowel movement within the subsequent 24 h), and time to hospital discharge (for participants admitted to or treated in hospital, based on day of discharge, provided that there was no readmission for *C difficile* infection before test of cure visit). Participants who were lost to follow-up, discontinued the study, or received other treatment for *C difficile* infection before test of cure were regarded as having treatment failure. Sustained clinical response for each participant was centrally assessed on the basis of investigator-determined cure at test of cure and recurrence.

The secondary objectives were assessment of the safety and tolerability of ridinilazole compared with that of vancomycin (occurrence of adverse and serious adverse events), plasma and faecal concentrations of ridinilazole, and health status (assessed by participant-reported responses to the EQ-5D-3L questionnaire).

Safety assessments comprised physical examination, vital signs (eg, blood pressure, pulse, weight, and body temperature), clinical laboratory evaluations (eg, haematology, coagulation, clinical chemistry, and urinalysis), and electrocardiograms. Safety was assessed in all participants who received the study medication from the time of consent until the last study assessment, regardless of their response to treatment. We coded adverse events using the Medical Dictionary for Regulatory Activities (MedDRA; version 17.0). Treatment-emergent adverse events were defined as adverse events that started or increased in severity at the time of or after administration of the first dose of study medication.

Prespecified exploratory objectives of this study comprised qualitative and quantitative effects on the bowel microbiota of participants identified by 16sRNA sequencing; effects of treatment on calprotectin, lactoferrin, tumour necrosis factor-α (TNFα), interleukin 1β, interleukin 1 receptor antagonist, interleukin 8, and interleukin 23 in faecal samples over the course of therapy; and the effects of treatment on the microbiology of faecal samples voided over the course of therapy. Microbiology assessments included *C difficile* spore counts and the culture, isolation, and quantification of *C difficile* and vancomycin-resistant enterococci. *C difficile* isolates underwent ribotyping and susceptibility testing with an antibiotic panel that comprised fidaxomicin, moxifloxacin, vancomycin, metronidazole, rifampicin, rifaximin, chloramphenicol, clindamycin, tigecycline, ridinilazole, imipenem, and linezolid. Data on exploratory endpoints will be reported at a later date.

### Statistical analysis

The primary efficacy analysis was done on a modified intention-to-treat (ITT) population (all randomly assigned participants who had a diagnosis of *C difficile* infection with free toxin in stool and received one or more doses of the study medication), in view of evidence that patients with free toxin in stool are highly likely to have true *C difficile* infection^23^, ^24^, ^25^ and in keeping with *C difficile* infection diagnostic guidelines.^26^, ^27^ We tested the robustness of the primary outcome through analysis of the ITT population (randomly assigned participants who received one or more doses of the study medication) and per-protocol population (participants in the modified ITT population who had not violated any inclusion or exclusion criteria or deviated from the protocol in a way that could affect their efficacy assessments). We analysed safety in all randomly assigned participants who received at least one dose of study drug.

For the primary endpoint of sustained clinical response, we determined that with 100 participants there would be 82% power of concluding non-inferiority of ridinilazole versus vancomycin with a one-sided test at the 5% significance level and a non-inferiority margin of 15%, assuming 1:1 randomisation of 100 participants and sustained clinical response rates of 55% for vancomycin and 65% for ridinilazole. For the secondary endpoint of clinical response at test of cure, we determined that there would be 80% power of concluding non-inferiority of ridinilazole versus vancomycin with a one-sided test at the 5% significance level and a non-inferiority margin of 15%, assuming 1:1 randomisation of 100 participants and clinical response rates of 90% for both vancomycin and ridinilazole.

For the primary efficacy analysis, we calculated the sustained clinical response rate for each treatment group. We calculated the difference in clinical response rates between treatment groups (ridinilazole versus vancomycin) and the 90% two-sided CI for this difference on the basis of the stratified (by age group and history of recurrent *C difficile* infection) Miettinen and Nurminen method.^28^ Non-inferiority was considered to have been shown if the lower limit of the two-sided 90% CI for the difference between treatment proportions (ridinilazole versus vancomycin) was more than −0·15. We did a sensitivity analysis, excluding participants whose subsequent *C difficile* free toxin test result at recurrence was negative.

In addition, we analysed the proportion of patients achieving a sustained clinical response using a logistic regression model, with treatment, age category, and history of recurrent *C difficile* infection in the past 12 months (no episodes or one to three episodes) as factors. For the secondary efficacy analysis, we analysed cure at test of cure for the modified ITT, ITT, and per-protocol populations using the same methods as for the primary efficacy analysis. We summarised the rate of recurrence of *C difficile* infection for patients with a clinical response at test of cure. We analysed both time to resolution of diarrhoea and time to hospital discharge using a Cox proportional hazards model, with treatment, age category, and history of recurrent *C difficile* infection in the past 12 months as factors. We constructed Kaplan–Meier curves for each treatment group and used them to estimate median time to resolution of diarrhoea and median time to hospital discharge for days 1–12. We did all the statistical analyses with SAS (version 9.1 or higher).

We did subgroup analyses for the primary efficacy variable (sustained clinical response) and the secondary endpoint of clinical response at test of cure for the following predefined subgroups of the modified ITT population: age group (<75 years *vs* ≥75 years, and <65 years *vs* ≥65 years), history of recurrent *C difficile* infection in the past 12 months (no occurrences *vs* one to three previous occurrences), baseline severity based on modified European Society of Clinical Microbiology and Infectious Diseases (ESCMID) criteria (non-severe *vs* severe),^8^ presence of ribotype 027 strain, use of concomitant antibiotics at baseline, and hospital status (ie, inpatient or outpatient) at the time of randomisation.

This study has been registered with ClinicalTrials.gov, number NCT02092935.

### Role of the funding source

The Wellcome Trust, which co-funded this study, had no role in the design of the study, data analysis or interpretation, or writing of this report. The sponsor and other co-funder of the study was Summit Therapeutics, which was responsible for study design, data collection, data analysis, and writing of the manuscript. The corresponding author had full access to all study data and was responsible for the decision to submit the report for publication.

## Results

**Figure 1:**
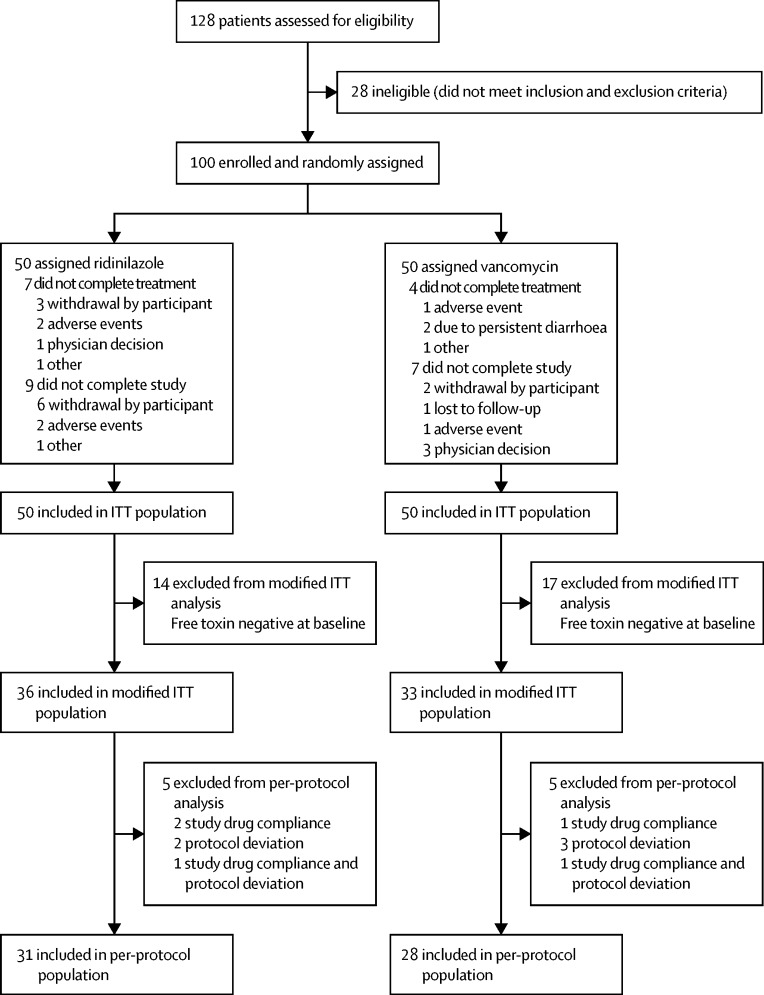
Trial profile ITT=intention-to-treat.

100 participants were recruited and randomly assigned (n=50 per treatment group) to receive at least one dose of study medication; all participants were included in the ITT and safety populations. The modified ITT population included 69 participants (n=36 in the ridinilazole group; n=33 in the vancomycin group) with a diagnosis of *C difficile* infection confirmed by the presence of free toxin. 59 participants (n=31 in the ridinilazole group; n=28 in the vancomycin group) were included in the per-protocol population (figure 1). 89 of 100 participants completed study treatment, with 11 discontinuing study treatment early. 84 participants completed the study, with 16 withdrawing from the study prematurely. The number of participants who completed the study was similar across both treatment groups: 41 in the ridinilazole group and 43 in the vancomycin group (figure 1).

There were no notable imbalances in baseline characteristics between treatment groups that could affect the assessment of efficacy or safety (table 1). The majority of participants were younger than 65 years and white, with no history of *C difficile* infection in the preceding 12 months, and the number of participants who had at least one previous episode was similar across the treatment groups (table 1). Disease severity was similar across treatment groups, with most participants having mild *C difficile* infection. Of the remainder, 18% in the ridinilazole group and 20% in the vancomycin group had moderate disease, whereas 14% in the ridinilazole group and 18% in the vancomycin group had severe *C difficile* infection (table 1). Severity could not be assessed in 4% of participants in each group because of missing data.

**Table 1:** Baseline characteristics

		**Ridinilazole (n=50)**	**Vancomycin (n=50)**
Age (years)			
	Mean (SD)	57·8 (17·43)	55·9 (20·29)
	Median (range)	59·0 (22–86)	61·0 (21–89)
Age group			
	<65 years	31 (62%)	28 (56%)
	65–75 years	11 (22%)	13 (26%)
	≥75 years	8 (16%)	9 (18%)
Sex			
	Male	21 (42%)	13 (26%)
	Female	29 (58%)	37 (74%)
Race			
	White	47 (94%)	45 (90%)
	Native American or Alaska Native	1 (2%)	1 (2%)
	African American	2 (4%)	2 (4%)
	Multiple	0	2 (4%)
History of recurrent *Clostridium difficile* infection in past 12 months			
	None	43 (86%)	44 (88%)
	One previous occurrence	5 (10%)	4 (8%)
	Two previous occurrences	2 (4%)	2 (4%)
Previous antibiotic treatments for *C difficile* infection			
	Vancomycin	4 (8%)	1 (2%)
	Metronidazole	10 (20%)	11 (22%)
	Vancomycin and metronidazole	5 (10%)	8 (16%)
Disease severity*			
	Mild	32 (64%)	29 (58%)
	Moderate	9 (18%)	10 (20%)
	Severe	7 (14%)	9 (18%)
	Missing	2 (4%)	2 (4%)
Presence of ribotype 027 strain		7 (14%)	5 (10%)

* Disease severity was assessed by use of the modified European Society of Clinical Microbiology and Infectious Diseases criteria used in the phase 3 studies of fidaxomicin.^8^ Severity categories were mild (<6 unformed bowel movements per day or white blood cell [WBC] count ≤12 000 μL), moderate (6–9 unformed bowel movements per day or WBC 12 001–15 000 μL), and severe (≥10 unformed bowel movements per day or WBC counts >15 000 μL).

**Figure 2:**
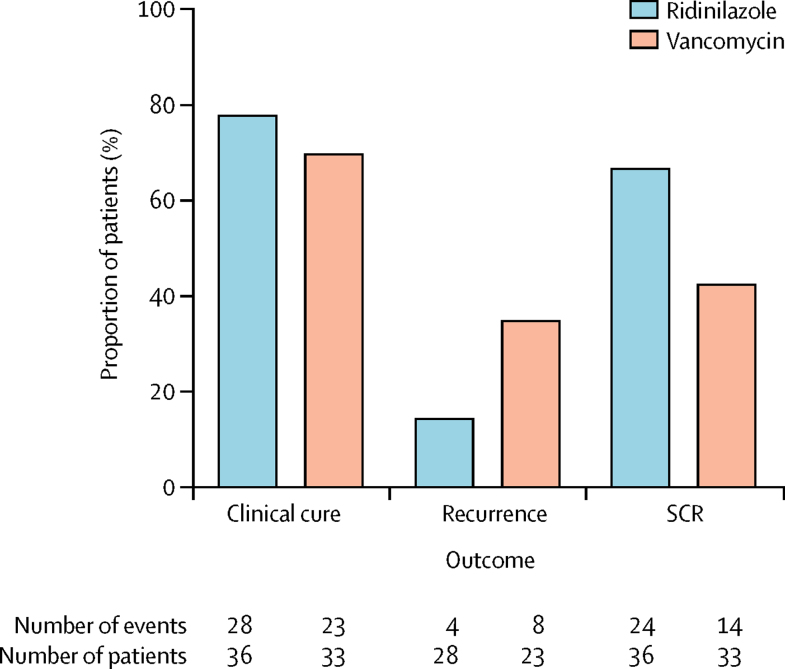
Efficacy analysis in the modified intention-to-treat population SCR=sustained clinical response.

In the modified ITT population, 24 (66·7%) of 36 patients in the ridinilazole group versus 14 (42·4%) of 33 in the vancomycin group had a sustained clinical response (figure 2; table 2). The estimated treatment difference was 21·1% (90% CI 3·1–39·1; p=0·0004), which showed non-inferiority of ridinilazole versus vancomycin (table 2). Because the 90% CI lies entirely above zero, ridinilazole was superior to vancomycin as shown at the 10% level (two-sided test). In the modified ITT population, one participant in the ridinilazole group had a negative free toxin result at the time of diagnosis of recurrence. Exclusion of this participant in a sensitivity analysis resulted in sustained clinical responses in 24 (68·6%) of 35 patients in the ridinilazole group (treatment difference 22·6%, 90% CI 4·6–40·6). Efficacy analyses in the ITT and per-protocol populations supported the finding of non-inferiority, with the superiority of ridinilazole versus vancomycin on sustained clinical response also being shown in the per-protocol population (appendix).

**Table 2:** Primary and secondary efficacy outcomes in the modified intention-to-treat population

		**Ridinilazole (n=36)**	**Vancomycin (n=33)**	**Treatment difference (90% CI)**	**p value**
Sustained clinical response		24 (66·7%)	14 (42·4%)	21·1% (3·1 to 39·1)*	0·0004†
Clinical response at test of cure		28 (77·8%)	23 (69·7%)	8·3% (−9·3 to 25·8)*	..
Time to resolution of diarrhoea					
	Mean (SD), days	4·6 (2·52)	5·1 (3·15)	..	..
	Median time (IQR), days	4·0 (3·0 to 6·0)	5·0 (3·0 to 10·0)	1·19 (0·76 to 1·87)‡	..
Time to hospital discharge					
	Number of inpatients at randomisation	7 (19·4%)	9 (27·2%)	..	..
	Mean time (SD), days	4·6 (3·29)	3·7 (3·01)	..	..
	Median time (IQR), days	5·0 (2·0 to 10·0)	7·0 (2·0 to 8·0)	0·99 (0·34 to 2·91)‡	..

* Treatment difference with 90% CI based on the stratified (by age group and history of recurrent *Clostridium difficile* infection) Miettinen and Nurminen method.

† p value is based on the Wald test and a non-inferiority margin of 15%.

‡ Hazard ratio and 90% CI obtained from a Cox proportional hazards model with treatment, age category (<75 years or ≥75 years), and history of recurrent *C difficile* infection in the past 12 months (no occurrences or one to three previous occurrences) as factors.

**Figure 3:**
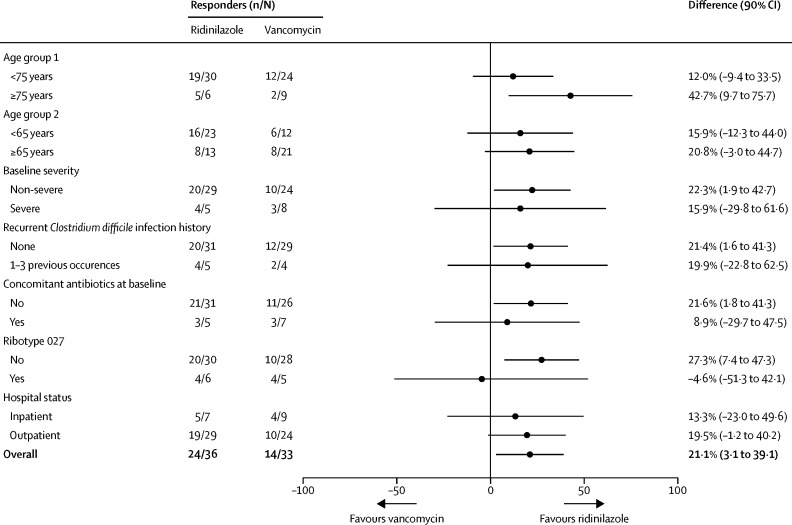
Subgroup analysis of sustained clinical response in the modified intention-to-treat population

In prespecified subgroup analyses, ridinilazole was generally associated with more sustained clinical responses than was vancomycin, with differences of 42·7% (90% CI 9·7 to 75·7) for patients older than 75 years, 15·9% (−29·8 to 61·6) for those with for severe disease, 19·9% (−22·8 to 62·5) for those with previous episodes of *C difficile* infection, and 8·9% (−29·7 to 47·5) for those who were receiving concomitant antibiotics at baseline (figure 3). For the presence of ribotype 027 at baseline, the difference in response was −4·6% (90% CI −51·3 to 42·1; figure 3). In the modified ITT population, 11 participants (six in the ridinilazole group and five in the vancomycin group) were confirmed as having a ribotype 027 isolate at baseline (figure 3). At the test of cure visit, five of six participants in the ridinilazole group and four of five in the vancomycin group were considered to have been cured clinically. One recurrence was noted in the ridinilazole group and no recurrences recorded in the vancomycin group among patients with ribotype 027 infection. Because of the small numbers of participants with this strain, firm conclusions on the relative efficacies of the study drugs for this subgroup cannot be drawn.

In the modified ITT population, 28 (77·8%) of 36 patients in the ridinilazole group versus 23 (69·7%) of 33 in the vancomycin group had a clinical response at test of cure (figure 2; table 2). The difference between treatment groups was 8·3% (90% CI −9·3 to 25·8), which shows the non-inferiority of ridinilazole versus vancomycin. Among participants considered to have treatment failure, 13·9% (five of 36) in the ridinilazole group and 21·2% (seven of 33) in the vancomycin group were considered by investigators to have treatment failure either at the test of cure visit or during study drug dosing. All remaining participants were considered to have treatment failure at test of cure for other reasons, such as discontinuation from the study. This result was corroborated by similar analysis of the per-protocol population, but we did not show non-inferiority in the intention-to-treat population (appendix).

The median time to resolution of diarrhoea in the modified ITT population was 4·0 days (IQR 3·0–6·0) for patients in the ridinilazole group compared with 5·0 days (3·0–10·0) for those in the vancomycin group (hazard ratio [HR] 1·19, 90% CI 0·76–1·87; table 2). In a post-hoc analysis, diarrhoea resolved by day 6 in a greater proportion of patients in the ridinilazole group than in the vancomycin group (77·8% [28 of 36] *vs* 63·6% [21 of 33]). By the test of cure visit, diarrhoea had not resolved in one (2·8%) of 36 patients in the ridinilazole group versus three (9·1%) of 33 in the vancomycin group. The median time to hospital discharge was similar for the two groups (table 2).

Recurrence was analysed only in participants who were considered clinically cured at test of cure. 51 participants in the modified ITT population met this criterion (28 in the ridinilazole group, 23 in the vancomycin group). One participant (who was randomly assigned to receive vancomycin) did not report a recurrence but discontinued the study before day 40 and therefore had to be considered to have failure for sustained clinical response. Recurrence (either unconfirmed or confirmed by a diagnosis including the presence of free toxin in stool) was recorded in four (14·3%) of 28 participants in the ridinilazole group versus eight (34·8%) of 23 in the vancomycin group (treatment difference −16·2%, 90% CI −35·5 to 3·0) in the modified ITT population (appendix; figure 2). Similar results were observed with the ITT and per-protocol populations (appendix).

We detected little systemic exposure of ridinilazole, with median concentrations of 0·00 ng/mL (range 0·00–0·59) on day 1, 0·09 ng/mL (0·00–1·31) on day 5, and 0·14 ng/mL (0·00–1·06) on day 10, 3–5 h after the first active dose of the day. Faecal concentrations were high, with mean concentrations of 1298 μg/g (SD 1302) on day 5 and 1373 μg/g (1390) on day 10.

Ridinilazole was well tolerated, with an adverse event profile similar to that of vancomycin (table 3). Of all participants, 82% in the ridinilazole group and 80% in the vancomycin group reported treatment-emergent adverse events, whereas 16% in the ridinilazole group and 18% in the vancomycin group reported treatment-emergent serious adverse events. One participant in the vancomycin group discontinued treatment because of a gastrointestinal disorder (moderate haematemesis) that was deemed to be related to the study drug. Three participants had serious adverse events that were considered likely to be related to the study drug: one participant in the ridinilazole group had hypokalaemia, one participant in the vancomycin group had septic shock (this was the same participant who had moderate haematemesis leading to discontinuation from the study), and one participant in the vancomycin group had elevated liver enzymes and diarrhoea. Two deaths in the vancomycin group (one due to hepatic cancer and one due to malnutrition) occurred during follow-up, with neither considered to be related to the study medication (table 3). Most adverse events occurred in the gastrointestinal system organ class for both treatment groups (40% in ridinilazole recipients and 56% in vancomycin recipients; table 3). A greater proportion of participants receiving ridinilazole than those receiving vancomycin had adverse events related to metabolism and nutrition disorders, and to respiratory, thoracic, and mediastinal disorders (table 3).

**Table 3:** Adverse events

		**Ridinilazole (n=50)**	**Vancomycin (n=50)**
**Summary**			
Total number of adverse events		180	183
TEAEs		41 (82%)	40 (80%)
Drug-related TEAEs		8 (16%)	10 (20%)
Severe TEAEs		8 (16%)	6 (12%)
Severe drug-related TEAEs		2 (4%)	1 (2%)
Deaths		0	2 (4%)
SAEs		8 (16%)	9 (18%)
Treatment-emergent SAEs		8 (16%)	9 (18%)
Drug-related treatment-emergent SAEs		1 (2%)	2 (4%)
Discontinuations because of TEAEs		2 (4%)	1 (2%)
Discontinuations because of drug-related TEAEs		0	1 (2%)
System organ class preferred term*			
Gastrointestinal disorders		20 (40%)	28 (56%)
	Nausea	10 (20%)	9 (18%)
	Abdominal pain	6 (12%)	10 (20%)
	Abdominal distension	5 (10%)	5 (10%)
	Vomiting	5 (10%)	8 (16%)
	Flatulence	4 (8%)	2 (4%)
	Diarrhoea	0	5 (10%)
General disorders and administration site conditions		12 (24%)	10 (20%)
	Asthenia	3 (6%)	2 (4%)
	Oedema (peripheral)	2 (4%)	3 (6%)
Infections and infestations		12 (24%)	12 (24%)
	Urinary tract infections	3 (6%)	2 (4%)
	Nasopharyngitis	2 (4%)	5 (10%)
Metabolism and nutrition disorders		11 (22%)	7 (14%)
	Decreased appetite	5 (10%)	4 (8%)
	Dehydration	3 (6%)	0
Nervous system disorders		10 (20%)	11 (22%)
	Headache	4 (8%)	5 (10%)
	Dizziness	3 (6%)	5 (10%)
Respiratory, thoracic, and mediastinal disorders		9 (18%)	3 (6%)
	Dyspnoea	4 (8%)	1 (2%)
Musculoskeletal and connective tissue disorders		6 (12%)	8 (16%)
	Back pain	1 (2%)	3 (6%)
	Pain in upper and lower extremities	1 (2%)	3 (6%)
Skin and subcutaneous tissue disorders		5 (10%)	5 (10%)
	Rash	3 (6%)	2 (4%)

TEAE=treatment-emergent adverse event. SAE=serious adverse event.

* Only TEAEs (all causalities) reported in three or more participants (≥6%) treated with either ridinilazole or vancomycin have been reported.

## Discussion

This phase 2 study showed the novel antimicrobial ridinilazole to be non-inferior to vancomycin with regard to both the primary endpoint of sustained clinical response and the secondary endpoint of clinical response at the test of cure visit in patients with *C difficile* infection confirmed by the presence of free toxin in stool. Moreover, ridinilazole was shown to be superior to vancomycin with regard to sustained clinical response in the primary analysis population. The overall adverse event profiles of ridinilazole and vancomycin were similar, with no safety signals being identified with ridinilazole. Oral administration of ridinilazole was associated with minimal systemic exposure, consistent with data in healthy volunteers at the same dose of 200 mg given twice a day for 10 days, suggesting that systemic exposure of ridinilazole is not increased in the presence of an inflamed gastrointestinal tract.^21^ No significant effect of ridinilazole on time to resolution of diarrhoea was evident.

Participants in this study were fairly representative of those who develop *C difficile* infection. We included patients older than 65 years, those with severe disease, and those with previous episodes of *C difficile* infection, all of whom are at increased risk of infection and of disease recurrence following treatment.^2^, ^4^, ^29^ Progressive deterioration in treatment outcomes has been documented in adults older than 40 years,^30^ and 93% of deaths related to *C difficile* infection in the USA in 2009 occurred in adults older than 65 years.^29^ Additional risk factors for *C difficile* infection include exposure to the health-care system, previous or current antibiotic use, being immunocompromised, and having underlying chronic comorbid conditions.^31^ Our finding that ridinilazole was associated with improved sustained clinical response compared with vancomycin in both the primary efficacy analysis and in secondary analyses of subgroups based on age, baseline severity, history of recurrence, baseline concomitant antibiotic use, and hospital (inpatient *vs* outpatient) status is encouraging. However, differences in sustained clinical response were not statistically significant in many of the subgroups, probably because of the small sample size, and these outcomes, along with others, will be further investigated in larger confirmatory studies.

We used the modified ITT population for the primary analysis on the basis of evidence that patients with a diagnosis confirmed by presence of free toxin are most likely to have true *C difficile* infection. Infection severity and clinical outcomes, including mortality and complications related to *C difficile* infection, are significantly worse in patients with *C difficile* infection defined by the presence of faecal toxin, as opposed to a toxigenic *C difficile* strain alone (eg, as defined by a toxin gene PCR-positive result).^23^, ^24^, ^25^ Such findings are likely to reflect the poor discriminatory power of molecular *C difficile* tests to distinguish colonisation from infection in elderly people with several comorbidities, who have a high chance of being colonised by toxigenic strains and thus might be falsely identified as having *C difficile* infection when they have diarrhoea due to another reason. Our focus on faecal-toxin-positive individuals is consistent with national and international *C difficile* infection diagnostic guidelines and, as such, adds rigour to our analyses.^26^, ^27^

Bacteria that are part of the normal gut microbiota directly confer resistance to colonisation by intestinal pathogens through, for example, bile salt metabolism, competition for nutrients, and production of inhibitory molecules, or indirectly through immune-mediated mechanisms.^32^ The superiority of ridinilazole compared with vancomycin with regard to sustained clinical response was driven by a marked reduction in recurrent *C difficile* infection, which is likely to be due to the highly selective activity of ridinilazole against *C difficile* and the absence of collateral damage to the microbiota during therapy.^33^ Sustained clinical response takes into consideration both initial cure rates and recurrence rates, and has therefore been a preferred measure of treatment outcomes for *C difficile* infection when comparing agents with similar initial clinical response rates.^34^

This study had some limitations. First, only 21 of the 33 sites recruited participants for the study. Although 14 sites recruited three or more participants, most of the recruitment (approximately 70%) was done across eight sites. Second, although study participants were reasonably representative of patients with *C difficile* infection, they were slightly younger and had milder disease. Third, the power calculation of the study was based on the assumption of 100 participants in the primary analysis population. However, although the primary analysis only included 69 participants, the study still met the primary endpoint, establishing non-inferiority and also showing statistical superiority at the prespecified 10% level. Fourth, recurrence was only monitored for 30 days after the end of treatment. Although this follow-up accords with similar phase 2 and phase 3 clinical trials,^7^, ^8^, ^22^ future studies should consider longer follow-up periods. Finally, 31 participants were not included in the primary analysis population because of a negative free toxin assay. This probably reflects, in part, the suboptimal sensitivity of free toxin assays resulting in false negative results, and poor specificity of molecular assays for detection of *C difficile* infection resulting in the potential for enrolment of colonised rather than infected participants. However, the rigour with which the modified ITT population was defined mitigates this limitation and supports the study's primary analysis and results.

Our findings suggest that ridinilazole has the potential to be an effective treatment of *C difficile* infection and support its assessment in larger phase 3 clinical trials.
