# Supplementary material for: Efficacy and safety of ridinilazole compared with vancomycin for the treatment of Clostridium difficile infection: a phase 2, randomised, double-blind, active-controlled, non-inferiority study
Source: Lancet Infect Dis. 2017 Jul;17(7):735–44. doi: 10.1016/S1473-3099(17)30235-9 (PMC5483507; doi:10.1016/S1473-3099(17)30235-9)
Supplement: Supplementary appendix [file mmc1.pdf]

THE LANCET
Infectious Diseases

Supplementary appendix

This appendix formed part of the original submission and has been peer reviewed. We post it as supplied by the authors.

Supplement to: Vickers RJ, Tillotson GS, Nathan R, et al, for the CoDIFy study group. Efficacy and safety of ridinilazole compared with vancomycin for the treatment of *Clostridium difficile* infection: a phase 2, randomised, double-blind, active-controlled, non-inferiority study. *Lancet Infect Dis* 2017; published online April 28. http://dx.doi.org/10.1016/S1473-3099(17)30235-9.

Appendix

Study design

This was a phase 2, multicentre, randomised, double-blind, active-controlled, parallel-group design study conducted in participants with a confirmed diagnosis of *Clostridium difficile* infection (CDI).

A total of 128 participants of either gender between the ages of 18 and 90 years at the time of informed consent were assessed for eligibility at 33 centres in the United States and Canada, with 100 participants with a diagnosis of CDI (ie, clinical signs and symptoms and a positive diagnostic test) being randomised. Participants were treated either as inpatients or as outpatients for part or all of their participation in the study. An outpatient was defined as a study participant who received any dose of the study drug in a non-acute care hospital setting, nursing home, or long-term acute care hospital, with the exception of the study drug received at a study site during a protocol-defined study visit.

Following the provision of informed consent and confirmation of eligibility at screening, participants were randomised to either of two arms and assigned a unique participant number:

- *Ridinilazole (formerly known as SMT19969) 200 mg two times a day (BID) orally over ten days (arm D1)*: For the entire ten-day treatment period, participants were asked to take four doses (capsules) of the study drug each day, with two containing 200 mg ridinilazole and two containing placebo. The first and third doses were SMT19969, while the second and fourth were placebo.
- *Vancomycin 125 mg four times a day (QID) orally over ten days (arm D2)*: For the entire ten-day treatment period, participants were asked to take four doses (capsules) of study drug each day, with all four capsules containing 125 mg vancomycin. Neither placebo nor ridinilazole were administered.

Due to the different dosing schedules between ridinilazole (BID) and the standard of care vancomycin (QID), placebo was used in arm D1 to match the QID dosing schedule of vancomycin and to preserve the blinded nature of the study. The study drugs, placebo, and packaging were manufactured such that participants and study site staff did not know the arm to which a participant had been assigned.

For participants treated as outpatients, the first dose of the study drug was administered under medical supervision in the clinic. Thereafter, study treatment could be self-administered, administered with the assistance of a caregiver, or given at a clinic. For inpatients, the study drug was administered by self-administration or by trained professional staff and recorded in the medical record. However, it was possible that some inpatients were discharged during the treatment period, in which case they self-administered the study treatment for the remainder of the treatment duration.

Overall study duration and follow-up

The study consisted of screening, treatment, and follow-up periods. The per-participant duration of study participation was approximately six weeks, consisting of ten days on treatment and 30 days of post-treatment follow-up. Any serious adverse events ongoing at study termination were planned to be followed until resolution or 30 days after the end-of-study (Day 40) visit, whichever came first.

Screening/baseline period and randomisation

Potential participants and their caregivers (where appropriate) were provided with information about the study and an informed consent document for their review. They also had the opportunity to discuss the study with a member of the study team before deciding whether they wished to enrol in the study. Only participants with signs and symptoms consistent with CDI, a confirmatory diagnostic test for *C. difficile*, and who met all inclusion/exclusion criteria (see next paragraph) were randomised to one of the two treatment regimens. Randomised treatment allocation was determined by a central, internet-based algorithm using a computer-generated random permutation procedure.

The randomisation was stratified by age of participant at time of enrolment into the study (under 75 years of age or 75 and over), history of recurrent CDI in the last 12 months (either none or one to three previous occurrences), and presence of free *C. difficile* toxin (A and/or B) at screening. All baseline examinations as well as faecal and blood

sampling were performed prior to administration of the first dose, although it was expected that the central laboratory data would not be available at the time of first dose with the study drug.

Treatment and follow-up
All participants received the study drug (QID) orally at approximately six hourly intervals for ten days. Participants were asked to take each dose of the study drug with approximately 60 mL of water. There were no food restrictions, although participants were asked to record in their study diary whether they took the study drug with or without food. Participants who were treated as outpatients and those who were discharged from the hospital prior to completion of the ten days of study drug therapy were asked to record the time of all self-administered doses in a participant study diary, which was also used to record any adverse events, the frequency and consistency of their stools (using the Bristol Stool Chart as a guide), clinical signs and symptoms of CDI, and their health and well-being. If hospitalized, participants were asked to complete the diary themselves if they were capable. These diary entries were reviewed with the participant by the site staff.

Participants were evaluated daily for safety while in the hospital and the assessments reviewed by the study site staff. Specific clinical assessments were obtained on Days 5, 10, 12, 25, and 40 post-first dose of the study drug (Appendix Table 1). Participants treated as outpatients adhered to the scheduled evaluations.

Unblinding
Allocation concealment was built into the interactive response technology (IXRS®) system. Emergency unblinding envelopes were provided to all sites, with each envelope containing a site-specific six-digit unblinding code. When emergency unblinding was required, individual sites were required to perform an unblinding action within the system, with entry of the code contained within the envelope being required to complete the unblinding action in order to receive the subject’s treatment assignment.

Study completion
Each participant received the study drug treatment for ten days, with Day 10 being defined as end of treatment (EOT) unless the study drug was prematurely discontinued, in which case EOT was defined as the last day the study medication was administered. A TOC evaluation occurred 48 hours after EOT (nominally Day 12) either as a site visit or as a phone follow-up. Subsequent protocol-defined assessment time points occurred between Days 13 and 39 (weekly telephone contact only), with in-person site visits on Day 25 and Day 40. The study was considered to have been completed after the last enrolled participant had completed the last defined follow-up visit and data monitoring had been completed prior to database lock and treatment unblinding.

Appendix Table 1: Schedule of study evaluations

Assessments ↓	Screening and baseline	Treatment period[a]			TOC	Post-treatment follow-up telephone contacts[a]	Post- treatment follow-up site visit	Recurrence visit[b]	Early termination visit	Day 30 post-treatment visit[a]
				EOT						
Study day →	Day −1	Day 1	Day 5 (±1)	Day 10 (+1)	Day 12 (+2)	Days 13–39	Day 25 (±3)	(Unscheduled)	(Unscheduled)	End of study Day 40 (±3)
Informed consent	X									
Inclusion/ exclusion	X									
Medical history	X									
Physical exam	X			X					X	X
Electrocardiogram	X[c]									
Vital signs[d]	X		X	X	X[a]				X	X
Central clinical lab tests (blood and urine)[e]	X		X	X	X[a]				X	
Local lab coagulation tests (PT/PTT/INR)	X[f]		X[f]	X[f]	X[a]				X[f]	
Drug concentration blood samples[g]		X	X	X					X	
Stool sample[h]	X		X	X	X[a]		X	X	X	X
Local lab test for *C. difficile*[i]	X							X		
Alere QUIK CHEK Test[j]	X							X		
Adverse events/serious adverse events[k]	X	X	X	X	X	X	X	X	X	X
Prior and concomitant medications	X	X	X	X	X	X	X	X	X	X
Signs and symptoms of CDI	X	X	X	X	X	X	X	X	X	X
Investigator determination of clinical response			X	X	X		X	X	X	X
EQ-5D-3L	X		X	X	X				X	X
Randomisation		X								
Study medication administration		X[l]	X	X						
Subject interview (CDI status)[a]	X	X	X	X	X	X	X	X	X	X
Stool count and characterisation[m]	X	X	X	X	X	X	X		X	X
Stool for drug concentration analysis	X		X	X					X	

[a]Participant interviews, which could have been by telephone, were conducted daily from treatment period Day 1 (Randomisation) through to the TOC (Day 12). If the evaluation was conducted by telephone, vital signs, central clinical lab tests (blood and urine), stool sample, and local lab coagulation tests (PT/PTT/INR) were not done. All participants were also contacted weekly thereafter up to 30 days post-treatment. Recurrent diarrhoea status was assessed during participant interviews, supplemented by review of the participant's personal records/study diary for the day, the time, and the quality of bowel movements. Participants who did not have a confirmed clinical response at the end of the treatment period were placed on standard of care and could have continued in the study or be withdrawn depending upon investigator/participant discretion. Participants with confirmed clinical response at the EOT visit and who subsequently experienced a recurrence were also placed on standard of care and continued in the study if investigator or participant chose to do so.
[b]Performed in participants who were considered to have a clinical response at the EOT visit and who had a recurrence prior to the Day-30 post-treatment visit.
[c]An electrocardiogram (ECG) was performed on all participants during the screening and baseline period. Additional follow-up ECGs were performed for any participant who experienced a cardiovascular-related significant medical event (eg, tachyarrhythmia) during the course of the study.

[d]Includes blood pressure, pulse, weight, and body temperature. Height was collected at baseline visit only.
[e]Includes biochemistry, haematology, serum pregnancy test (if applicable), vitamin E assay, and urinalysis.
[f]Coagulation parameters were monitored locally in those participants who were concomitantly receiving warfarin, heparin, or low-molecular weight heparin. Additional coagulation testing was performed as needed based on participants' clinical condition.
[g]Blood samples for study drug plasma concentration assessments were collected prior to dosing and four hours (±1) after the first dose on Day 1 and four hours (±1) after the first odd numbered dose on Day 5 and the final odd numbered dose on Day 10 (EOT visit) or at early termination visit if this occurred prior to Day 10.
[h]Stool samples were split into aliquots for the following additional tests: toxins A and B, ridinilazole drug concentration, microbiological testing, cytokines (calprotectin, lactoferrin, tumour necrosis factor (TNF)α, interleukin (IL)-1β, IL-1ra, IL-8, and IL-23), and microbiome analysis at the screening and baseline visit (DM1), Day 5, Day 10, Day 25, and Day 40.
[i]The use of any Food and Drug Administration(FDA)-cleared test to detect *C. difficile* could be utilized to determine eligibility for enrolment, including the Alere QUIK CHEK; enzyme immunoassays for *C. difficile* Toxin A (TcdA), *C. difficile* Toxin B (TcdB), or both toxins (TcdA/B); cell cytotoxicity neutralization assay; and nucleic acid amplification tests to detect the presence of either toxin genes or the pathogenicity locus (PaLoc).
[j]An Alere C. DIFF QUIK CHEK COMPLETE® rapid test was performed on stool from all subjects at screening. If this test was used to satisfy inclusion criterion 2, it was not repeated.
[j]Adverse events and serious adverse events were recorded on the case report form through Day-30 post-treatment.
[l]Study drug was administered after all other screening/baseline procedures were completed.
[m]Stool count includes number and description of all bowel movements within the given 24-hour day. For participants with rectal collection devices, volume was converted to number of unformed bowel movements (UBMs).

Sites could also use assays that were not FDA cleared as long as they were pre-approved by the study sponsor. The results of the assays' methods and performance characteristics (eg, sensitivity, specificity, cross-reactivity, precision, and reproducibility) of the assay(s) were required to be provided by the site for approval by the sponsor.

INR=international normalized ratio; PTT=partial thromboplastin time; PT=prothrombin time.

Inclusion criteria

To participate in this study, candidates were required to meet the following eligibility criteria at the time of screening:

- Ability to understand the purpose and risks of the study and to provide signed and dated informed consent and authorization to use protected health information in accordance with national and local subject privacy regulations.
- Male or female participants who were inpatients or outpatients at time of consent, 18 years of age or older (up to 90 years), and satisfied the diagnosis of CDI by clinical and laboratory criteria:
    - Diarrhoea, defined as a change in bowel habits, with greater than three unformed bowel movements (UBMs) or 200 mL or more unformed stool for participants having rectal collection devices in the 24 hours prior to randomisation;
    - Presence of either toxin A and/or B of *C. difficile* in the stool determined by any locally available Food and Drug Administration (FDA)-cleared test for *C. difficile* toxin, including the Alere C. DIFF QUIK CHEK COMPLETE® rapid test; enzyme immunoassays for *C. difficile* Toxin (TcdA), *C. difficile* Toxin B (TcdB), or both toxins (Tcd A/B); or cell cytotoxicity neutralization assay within 48 hours prior to randomisation. AND/OR
    - Presence of a toxigenic strain of *C. difficile* determined by a locally available FDA-cleared nucleic acid amplification test to detect the presence of either toxin genes or the pathogenicity locus (PaLoc) or non-FDA cleared tests within 48 hours prior to randomisation. Diagnostic tests that were not FDA-cleared could be considered for use provided results of the methods and performance characteristics (eg, sensitivity, specificity, cross-reactivity, reproducibility, etc.) of the assay(s) were provided by the site for review and approval by the study sponsor, and provided their performance characteristics were in line with those of comparable FDA-cleared tests.
- Participants with or without a history of previous episodes of CDI were eligible. However, participants should have had no more than three prior episodes of CDI in the previous 12 months and no previous episode within 30 days prior to study enrolment to be eligible.
- No more than 24 hours of prior antimicrobial treatment for the current episode of CDI with metronidazole, vancomycin, or fidaxomicin prior to screening.
- Female participants of childbearing potential and who were sexually active were informed (and were required to give consent) to use abstinence or two adequate and reliable methods of contraception (eg, barrier with additional spermicide foam or jelly, intrauterine device, or hormonal contraception). Females who were postmenopausal (at least one year without menses) were considered to not be of childbearing potential.
- Participants (both male and female) were required to agree to avoid conception during the treatment phase and until the end of their participation in the study.

Exclusion criteria

Candidates were excluded from study entry if any of the following conditions or criteria existed at the time of screening:

- Life-threatening or fulminant CDI with evidence of hypotension (systolic blood pressure less than 90 mmHg), septic shock, peritoneal signs or ileus, or toxic megacolon.
- History of inflammatory bowel disease (Crohn’s disease or ulcerative colitis).
- Pregnancy or lactation.
- Concurrent use of oral vancomycin, fidaxomicin, oral metronidazole, oral bacitracin, fusidic acid, rifamycins, tigecycline, nitazoxanide, or other drugs including herbal medications intended to treat CDI.
- Concurrent use of anti-diarrhoeals, anti-peristaltics, or any product with the potential to slow bowel movement.
- Participation in other clinical research studies using an investigational antibacterial agent within one month prior to screening.
- Inability in the opinion of the investigator or participant to discontinue aspirin, other non-steroidal anti-inflammatory drugs (NSAIDs), or oral vitamin E supplements from screening until Day 12 (TOC) or until the end of treatment if the participant discontinued from study.
- Use of $PY2_{12}$ inhibitors and inability to discontinue this treatment from screening until Day 12 (TOC) or until the end of treatment if the participant was discontinued from study.
- Known hypersensitivity or intolerance to ridinilazole, vancomycin, or their excipients.
- Therapy with a selective serotonin reuptake inhibitor (SSRI) and non-receipt of a stable dose of an SSRI.

Withdrawal from the study
Participants were free to withdraw from the study at any time. In addition, the investigator could decide, for reasons of clinical judgement, to withdraw a participant from study treatment if any of the following criteria were met:

- Any clinically relevant signs or symptoms which in the opinion of the investigator warranted participant withdrawal.
- Non-compliance with the protocol and/or study restrictions, as considered applicable by the investigator.
- Persistent diarrhoea, defined as more than three UBMs, in one day for two consecutive days after four days of therapy.

In all instances, the study sponsor was notified, and the date and reasons for the withdrawal were clearly stated in the participant's source data and the eCRF (case report form). If the randomisation code was broken for a participant, the date, time, and reason was recorded in the participant's source data.

If a participant was withdrawn or chose to withdraw anytime during the study, he or she was asked to return to the clinic for the early termination visit. Investigators followed withdrawn participants for any ongoing adverse events/serious adverse events until resolution or 30 days after the end-of-study (Day 40) visit, whichever came first.

Participants who withdrew following administration of one or more doses of the study drug were not replaced. Participants who were enrolled but for whom a confirmed diagnosis of CDI was not established were not randomised (treated with study drug). Once a participant was identified as having been randomised, that participant's randomisation number was not re-used.

Appendix Table 3: Efficacy outcomes (ITT and PP populations)

**SCR**	**Ridinilazole**	**Vancomycin**
	**n/N (%)**	**n/N (%)**
**ITT population**		
SCR success rate	32/50 (64·0)	25/50 (50·0)
Treatment difference (ridinilazole vs. vancomycin) and 90% CI[a]	13·5 (−1·8, 28·8)	
p value[b]	0·0015	
**PP population**		
SCR success rate	23/31 (74·2)	14/28 (50·0)
Treatment difference (ridinilazole vs. vancomycin) and 90% CI[a]	19·6 (0·5, 38·6)	
p value[b]	0·0007	
**Clinical Response at TOC**		
**ITT population**		
Cure	36/50 (72·0)	37/50 (74·0)
Treatment difference (ridinilazole vs. vancomycin) and 90% CI[a]	−1·6 (−16·2, 13·0)	
**PP population**		
Cure	26/31 (83·9)	22/28 (78·6)
Treatment difference (ridinilazole vs. vancomycin) and 90% CI[a]	6·3 (−11·1, 23·7)	

CI=confidence interval; ITT=intent to treat; PP=per protocol; SCR=sustained clinical response; TTROD=time to resolution of diarrhea

[a]90% CI is based on the stratified (age group and history of recurrent CDI factors) Miettinen and Nurminen method

[b]p value is based on the Wald test and a non-inferiority margin of 15%

Appendix Table 4: **Recurrence in participants with clinical response at the TOC visit**

**Analysis Population**	**Ridinilazole n/N* (%)**	**Vancomycin n/N* (%)**
**MITT Population**		
Confirmed recurrence by free toxin	3/28 (10·7)	7/23 (30·4)
Unconfirmed recurrence – negative free toxin	1/28 (3·6)	0/23 (0)
Unconfirmed recurrence – no toxin result	0/28 (0)	1/23 (4·3)
Discontinued	0/28 (0)	1/23 (4·3)
**ITT Population**		
Confirmed recurrence by free toxin	3/36 (8·3)	9/37 (24·3)
Unconfirmed recurrence – negative free toxin	1/36 (2·8)	1/37 (2·7)
Unconfirmed recurrence – no toxin result	0/36 (0)	1/37 (2·7)
Discontinued	0/36 (0)	1/37 (2·7)
**PP Population**		
Confirmed recurrence by free toxin	2/26 (7·7)	7/22 (31·8)
Unconfirmed recurrence – negative free toxin	1/26 (3·8)	0/22 (0)
Unconfirmed recurrence – no toxin result	0/26 (0)	1/22 (4·5)
Discontinued	0/26 (0)	0/22 (0)

*Only participants with a clinical response at the TOC visit are included.

CoDIFy Study Investigators

Ian Baird, MD
Remington-Davis, Inc.
1335 Dublin Road, Suite 106A
Columbus, OH 43215
United States

Amit Bhan, MD
39450 West 12 Mile Road
Novi, MI 48377
United States

Wayne Campbell, MD
210 East University Parkway
33rd Street Building, Suite 512
Baltimore, MD 21218
United States

Teena Chopra, MD
Harper University Hospital, 5 Hudson
3990 John Road
Detroit, MI 48201
United States

Kenneth Deck, MD
Alliance Research Centers
24012 Calle de la Plata, Suite 230
Laguna Hills, CA 92653
United States

Yoav Golan, MD
800 Washington Street
Holmes 4, Box #041
Boston, MA 02111
United States

Ian Gordon, MD, PhD
VA Long Beach Healthcare System
5901 East Seventh Street – Mail Code 11-112
Long Beach, CA 90822
United States

Ravi Kamepalli, MD
Regional Infectious Diseases and Infusion Center, Inc.
830 West High Street, Suite 255
Lima, OH 45801
United States

Sahil Khanna, MBBS
200 First Street Southwest
Rochester, MN 55905
United States

Christine Lee, MD
St. Joseph's Healthcare
50 Charlton Avenue East – L424

Hamilton, Ontario
L8N 4A6
Canada

Christopher Lucasti, DO
730 Shore Road
Somers Point, NJ 08244
United States

Benedict Maliakkal, MD
University of Rochester Medical Center
601 Elmwood Avenue, Box 646
Rochester, NY 14642
United States

Irene Minang, MD
The Duluth Clinic, Ltd.
400 East Third Street
Duluth, MN 55805
United States

Kathleen Mullane, DO
The University of Chicago Medicine
5841 South Maryland Avenue, MC 5065
Chicago, IL 60637
United States

Richard Nathan, DO
2900 Cortez Avenue
Idaho Falls, ID 83404
United States

Matthew Oughton, MD (replaced Andre Dascal, MD as Principal Investigator)
3755 Cote Ste. Catherine Road
Montreal, Quebec
H3T 1E2
Canada

Yves Pesant, MD
St-Jerome Medical Research Inc.
290 Montigny, Block F
St-Jerome, Quebec
J7Z 5T3
Canada

John Phillips, MD, PhD
North Mississippi Medical Center
589 Garfield Street, Suite 201
Tupelo, MS 38801
United States

John Pullman, MD
Mercury Street Medical Group, PLLC
300 West Mercury Street
Butte, MT 59701
United States

Paul Riska, MD
Montefiore Medical Center
111 East 210th Street
Bronx, NY 10467
United States

Camilla Saberhagen, MD
801 North 29th Street
Billings, MT 59101
United States

Christian Schrock, MD
3366 Oakdale Avenue North, Suite 520
Minneapolis, MN 55422
United States

Jonathan Siegel, MD
Internal Medicine Center
101 Memorial Hospital Drive, Suite 200
Mobile, AL 36608
United States

Alon Steinberg, MD
Ventura Clinical Research
1746 South Victoria Avenue, Suites 220 and 230
Ventura, CA 93003
United States

David Talan, MD
Olive View-UCLA Medical Center
14445 Olive View Drive, North Annex Building
Sylmar, CA 91342
United States

Stephen Tamang, MD
Regional Medical Clinic – Family Medicine
640 Flormann Street
Rapid City, SD 57701
United States

Michael Tan, MD
Summa Health System
75 Arch Street, Suite 105
Akron, OH 44304
United States

Karl Weiss, MD
5415 boul de l'Assomption
Montreal, Quebec
H1T 2M4
Canada

Thomas Welton, MD
720 Southwest Lane Street
Topeka, KS 66606

United States

Chia Wang, MD
1100 9th Avenue
Seattle, WA 98101
United States

Bruce Yacyshyn, MD
University of Cincinnati
Internal Medicine/Digestive Diseases
231 Albert Sabin Way
Cincinnati, OH 45267
United States

Jo-Anne Young, MD
University of Minnesota
420 Delaware Street Southeast
Mayo Memorial Building, MMC 250
Minneapolis, MN 55455
United States

Jonathan Zenilman, MD
Johns Hopkins Bayview Medical Center
5200 Eastern Avenue – MFL Center Tower, Suite 379
Baltimore, MD 21224
United States
